# Supplementary material for: Nanocomposite electrodes for high current density over 3 A cm−2 in solid oxide electrolysis cells
Source: Nat Commun. 2019 Nov 28;10:5432. doi: 10.1038/s41467-019-13426-5 (PMC6883038; doi:10.1038/s41467-019-13426-5)
Supplement: Supplementary file 1 — Supplementary Information [file 41467_2019_13426_MOESM1_ESM.pdf]

Supplementary Information

Nanocomposite electrodes for high current density over 3 A cm^{-2} in solid oxide electrolysis cells

Hiroyuki Shimada[1]*, Toshiaki Yamaguchi[1], Haruo Kishimoto[2], Hirofumi Sumi[1], Yuki Yamaguchi[1], Katsuhiro Nomura[1] and Yoshinobu Fujishiro[1]

[1]Inorganic Functional Materials Research Institute, Department of Materials and Chemistry, National Institute of Advanced Industrial Science and Technology (AIST), 2266-98 Anagahora, Shimo-shidami, Moriyama-ku, Nagoya, Aichi 463-8560, Japan

[2]Research Institute for Energy Conservation, Department of Energy and Environment, National Institute of Advanced Industrial Science and Technology (AIST), 1-1-1 Higashi, Tsukuba, Ibaraki 305-8565, Japan

* Corresponding author. E-mail: h.shimada@aist.go.jp

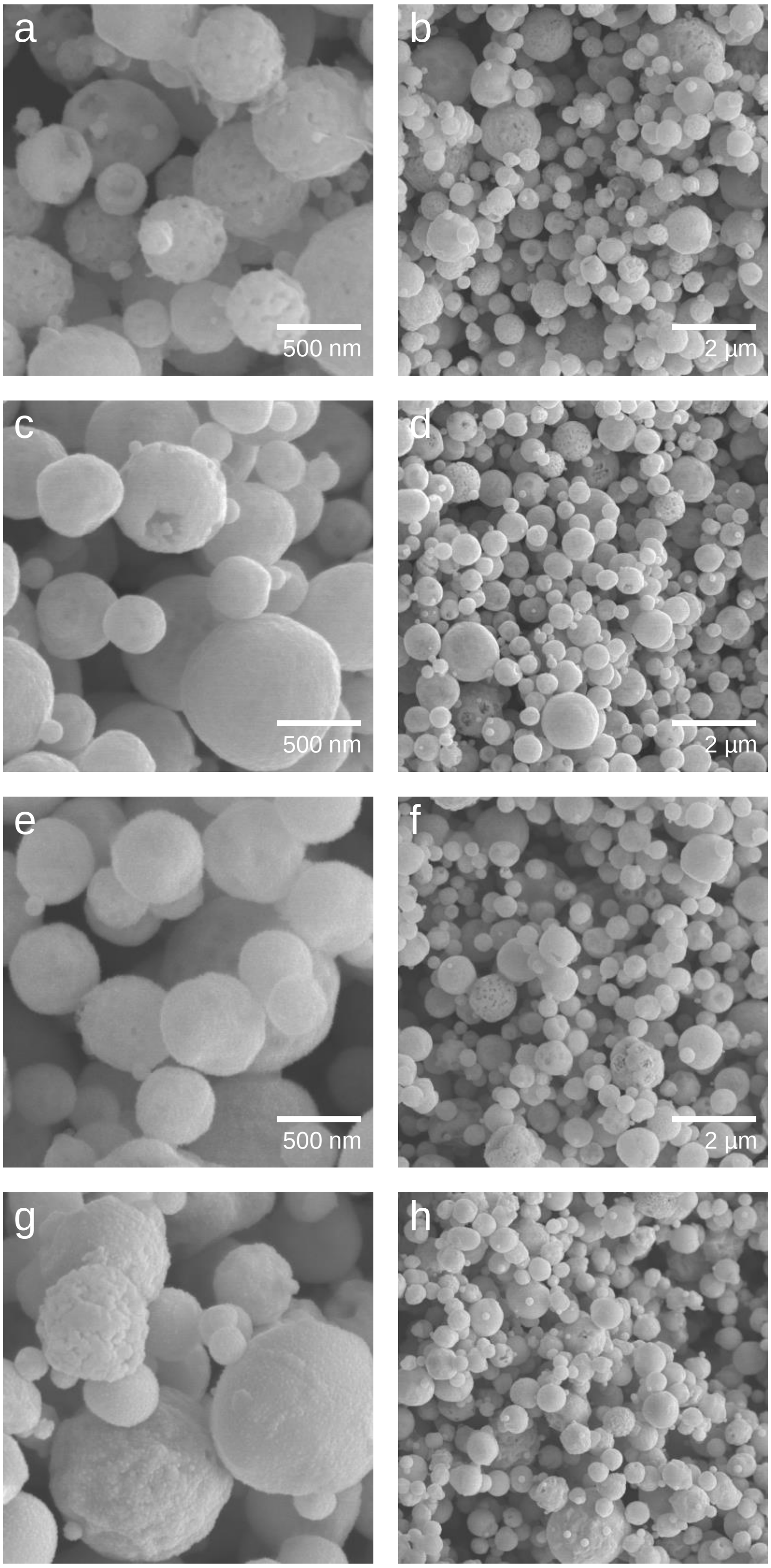

Supplementary Figure 1 FE-SEM images of SSC-SDC nanocomposite particles. **a**,**b** SSC-SDC(80:20), **c**,**d** SSC-SDC(70:30), **e**,**f** SSC-SDC(60:40), and **g,h** SSC-SDC(40:60).

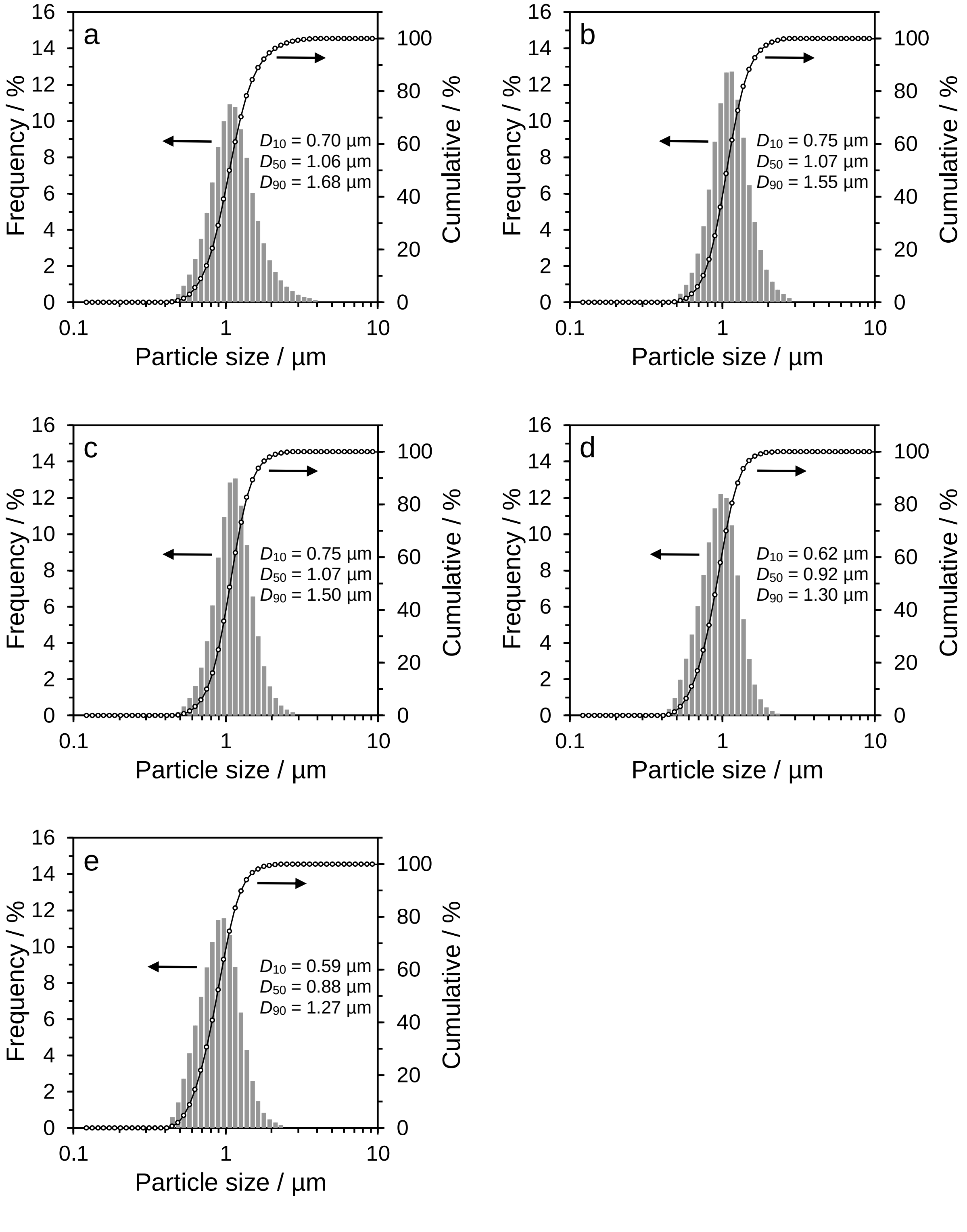

Supplementary Figure 2 Particle size distribution of SSC-SDC nanocomposite particles. **a** SSC-SDC(80:20), **b** SSC-SDC(70:30), **c** SSC-SDC(60:40), **d** SSC-SDC(50:50), and **e** SSC-SDC(40:60).

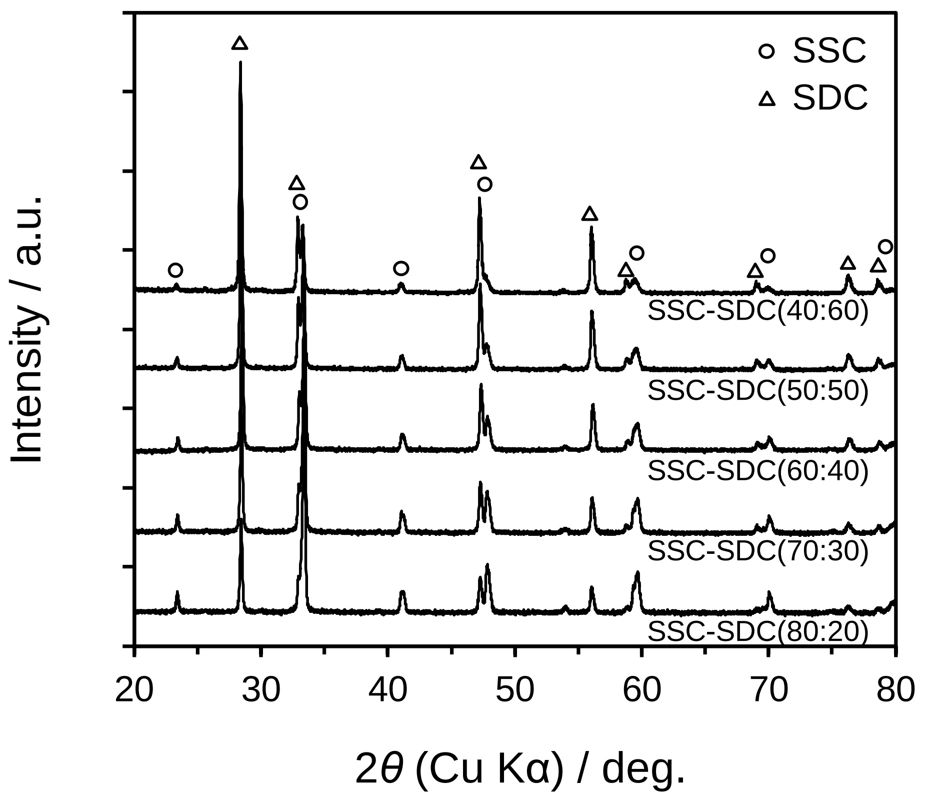

Supplementary Figure 3 XRD patterns for sintered SSC-SDC nanocomposite particles. Sintering condition of the particles was 950 °C for 1 h in air. XRD measurements were carried out at room temperature with Cu Kα radiation and with step size of 0.01°.

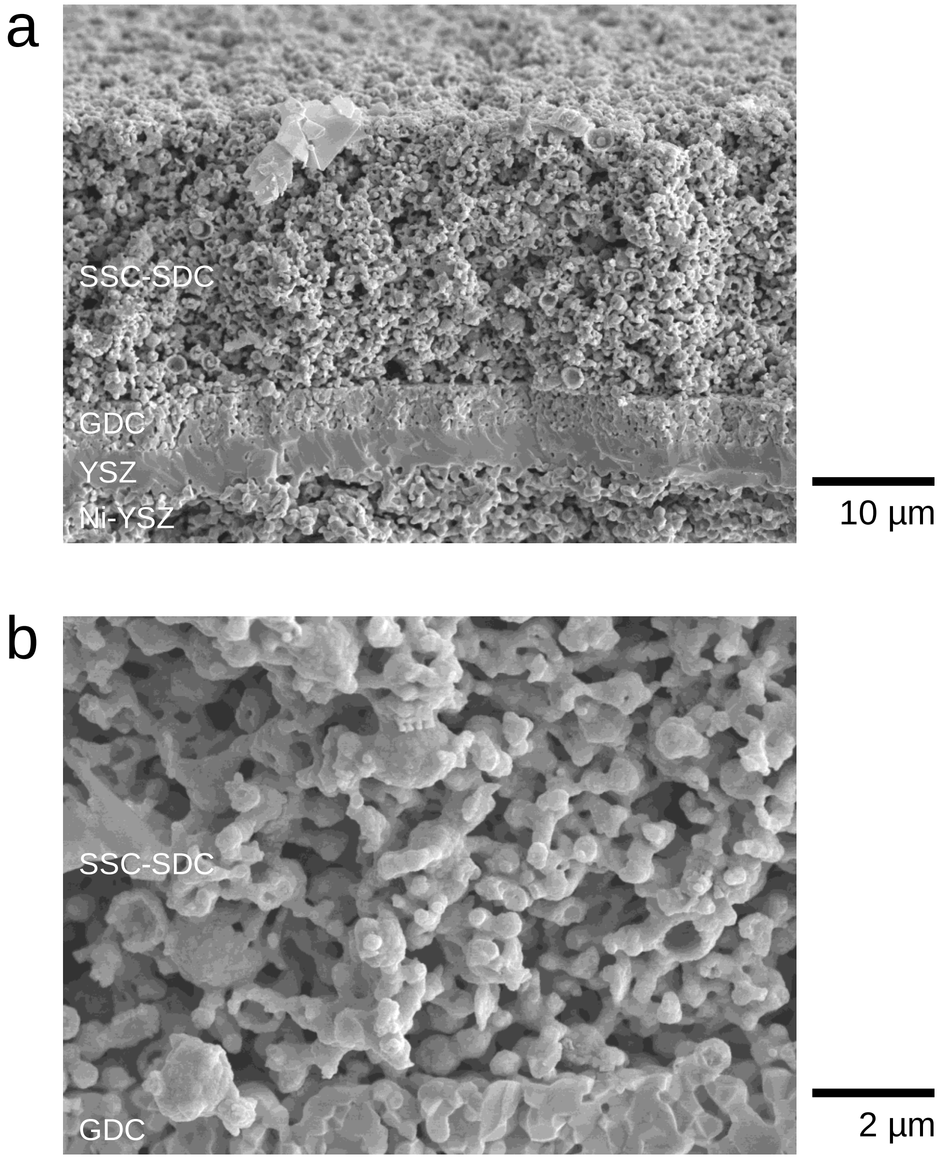

Supplementary Figure 4 Cross-sectional FE-SEM images of SSC-SDC(80:20) nanocomposite electrode. **a** SOEC consisting of SSC-SDC(80:20) nanocomposite electrode, GDC interlayer, YSZ electrolyte, and Ni-YSZ fuel electrode. **b** Enlarged image of SSC-SDC(80:20) nanocomposite electrode.

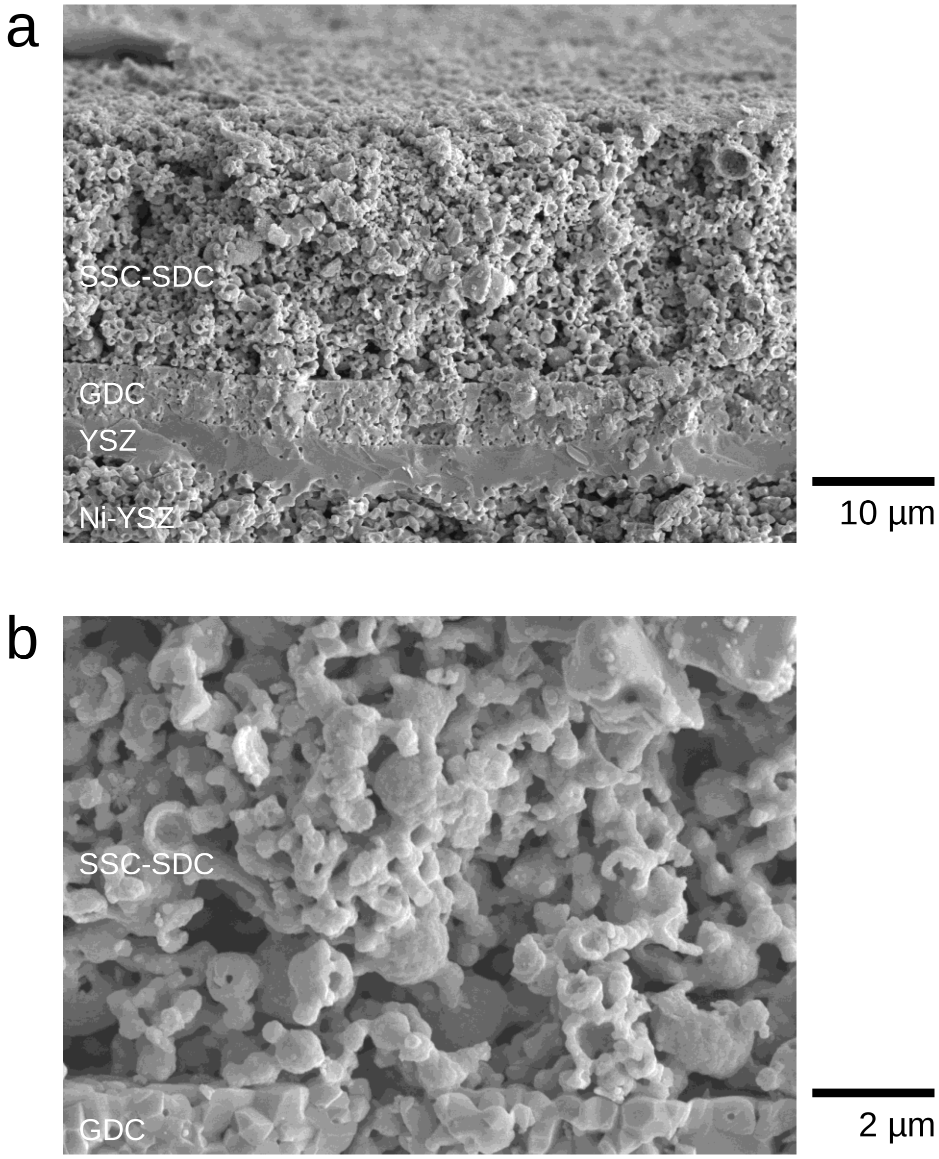

Supplementary Figure 5 Cross-sectional FE-SEM images of SSC-SDC(70:30) nanocomposite electrode. **a** SOEC consisting of SSC-SDC(70:30) nanocomposite electrode, GDC interlayer, YSZ electrolyte, and Ni-YSZ fuel electrode. **b** Enlarged image of SSC-SDC(70:30) nanocomposite electrode.

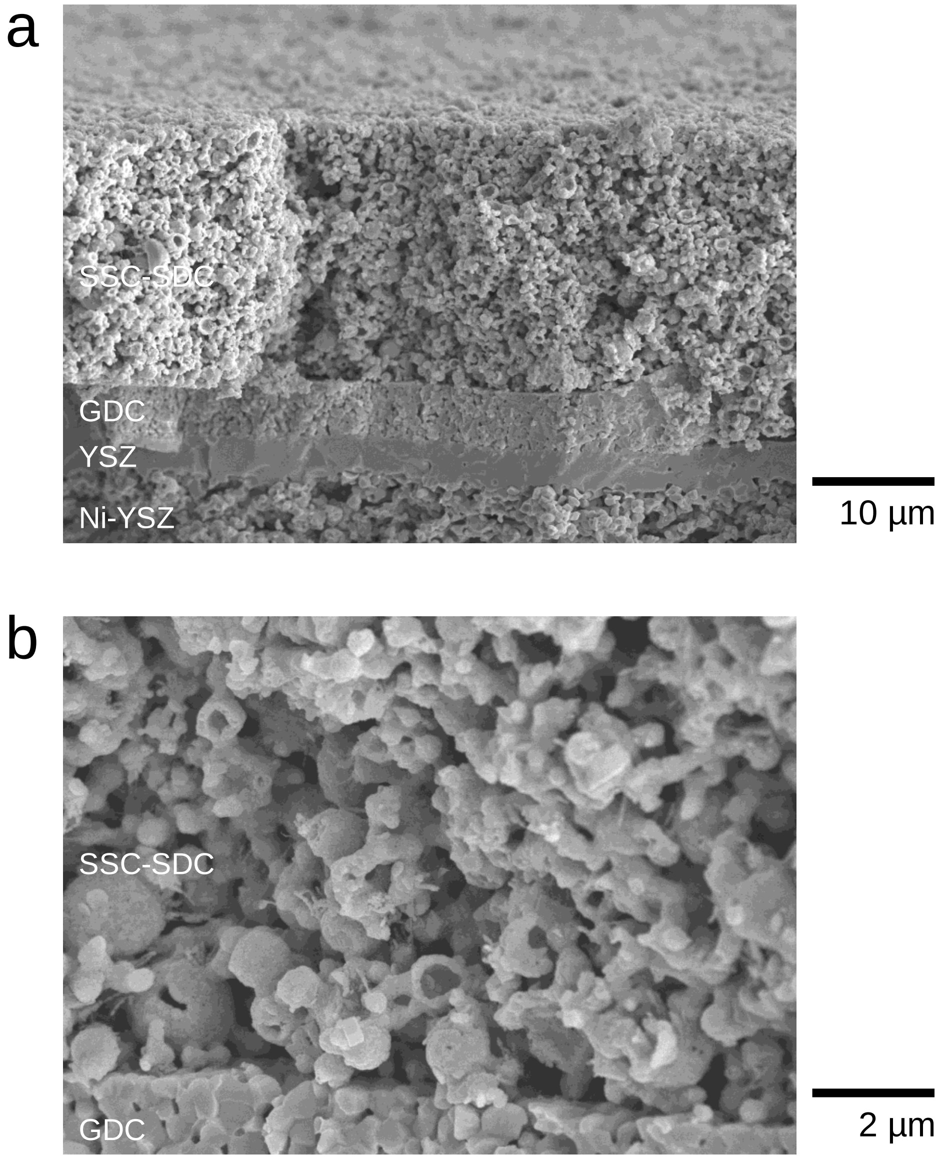

Supplementary Figure 6 Cross-sectional FE-SEM images of SSC-SDC(60:40) nanocomposite electrode. **a** SOEC consisting of SSC-SDC(60:40) nanocomposite electrode, GDC interlayer, YSZ electrolyte, and Ni-YSZ fuel electrode. **b** Enlarged image of SSC-SDC(60:40) nanocomposite electrode.

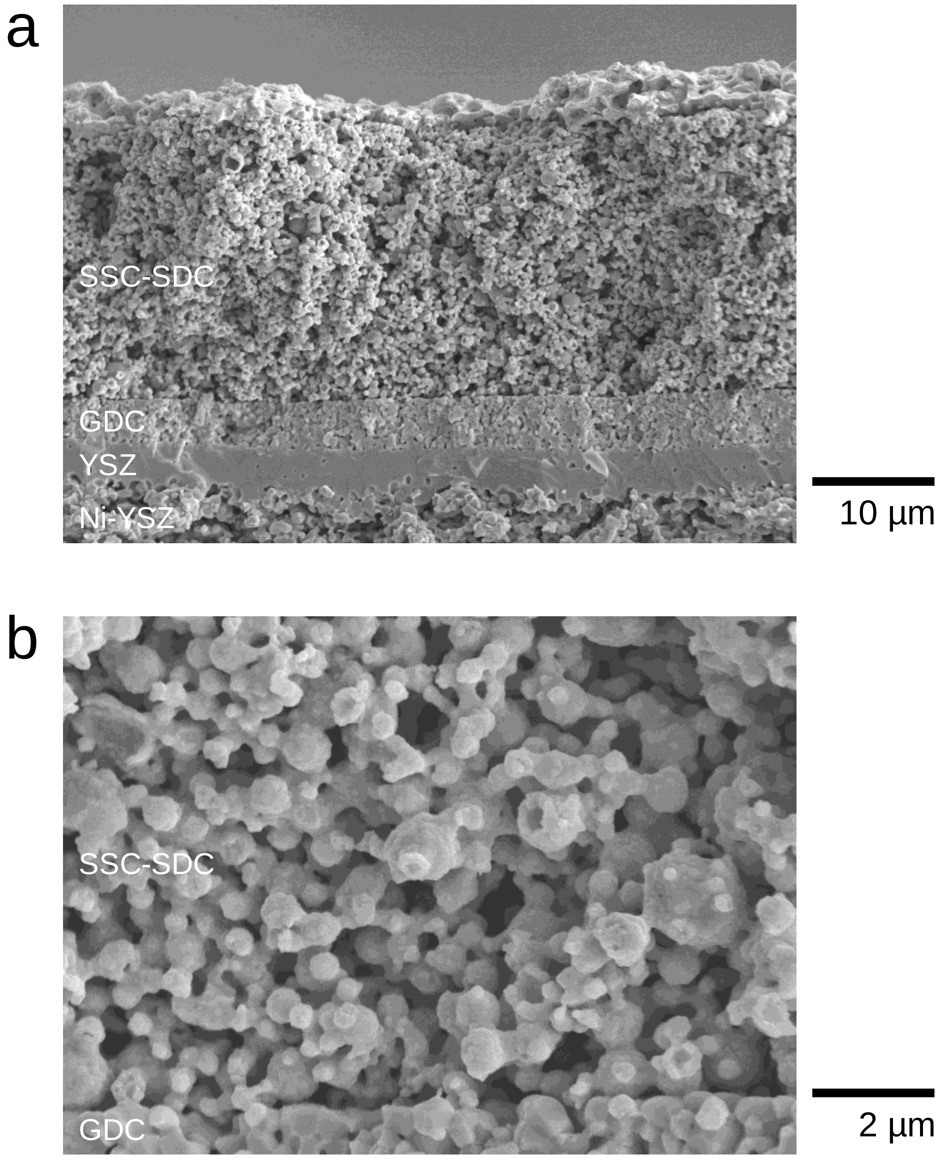

Supplementary Figure 7 Cross-sectional FE-SEM images of SSC-SDC(50:50) nanocomposite electrode. **a** SOEC consisting of SSC-SDC(50:50) nanocomposite electrode, GDC interlayer, YSZ electrolyte, and Ni-YSZ fuel electrode. **b** Enlarged image of SSC-SDC(50:50) nanocomposite electrode.

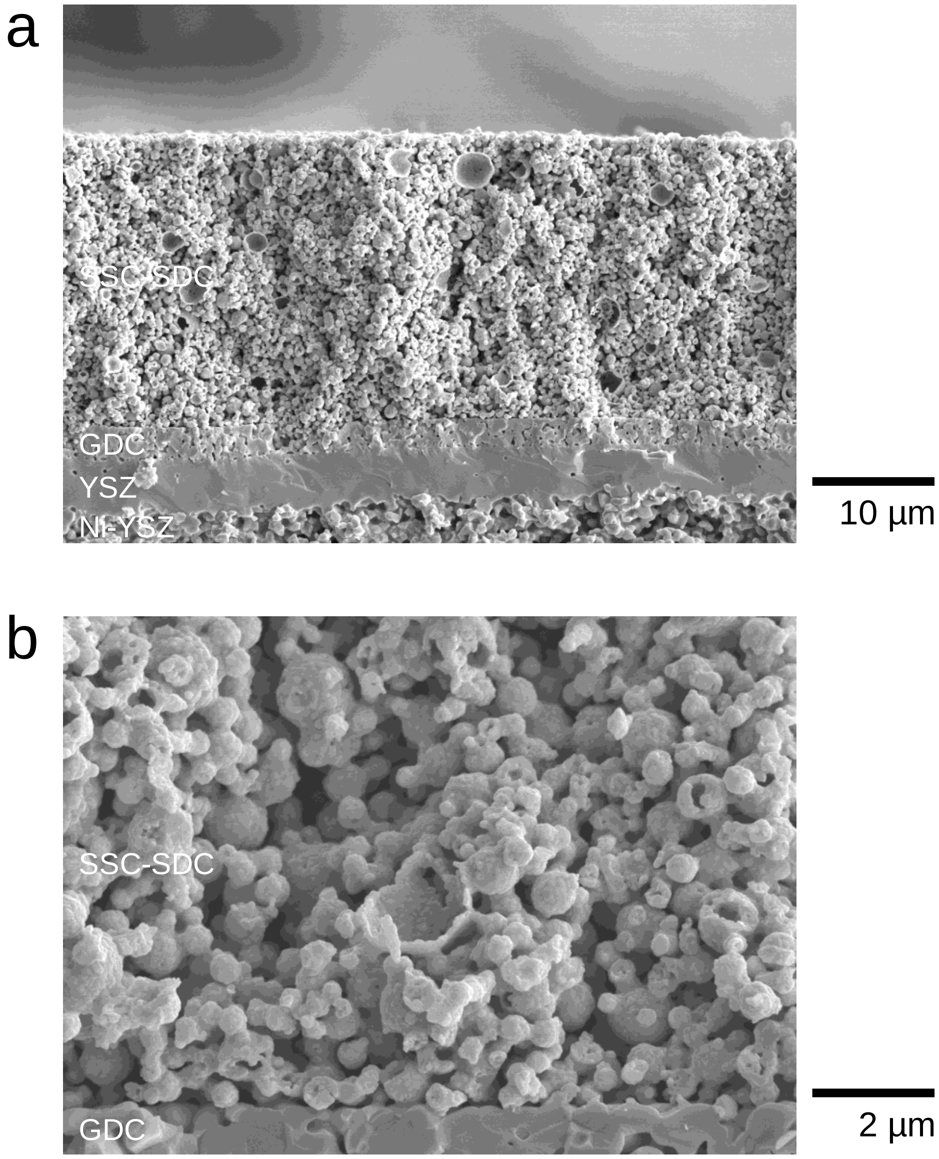

Supplementary Figure 8 Cross-sectional FE-SEM images of SSC-SDC(40:60) nanocomposite electrode. **a** SOEC consisting of SSC-SDC(40:60) nanocomposite electrode, GDC interlayer, YSZ electrolyte, and Ni-YSZ fuel electrode. **b** Enlarged image of SSC-SDC(40:60) nanocomposite electrode.

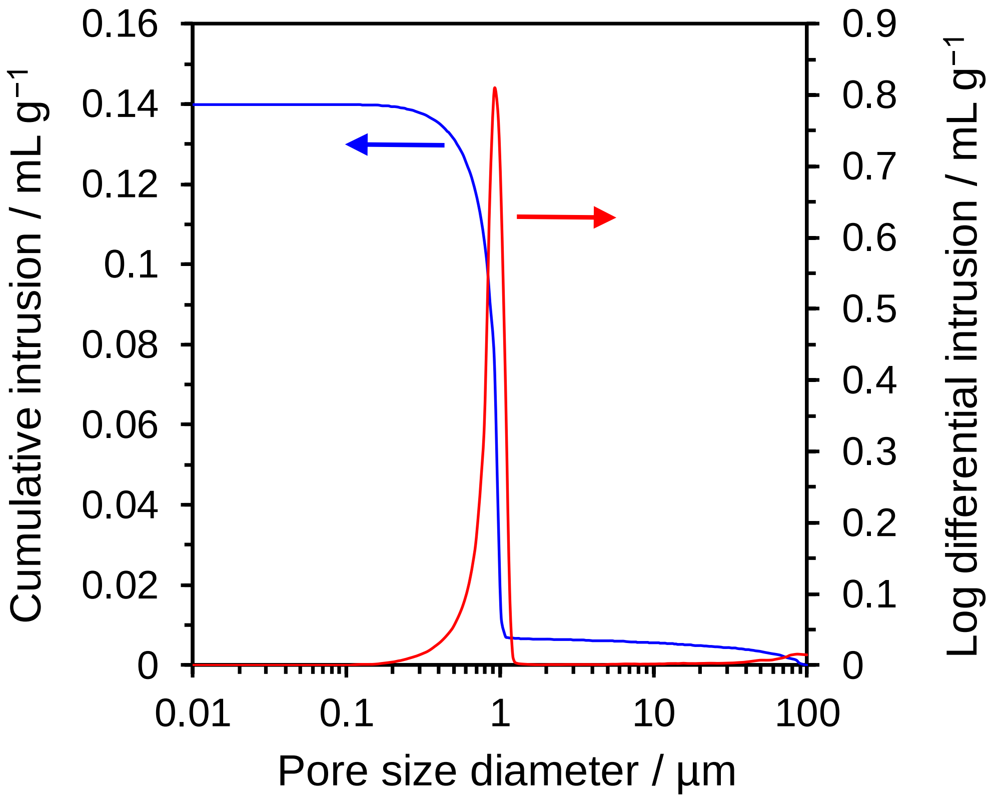

Supplementary Figure 9 Pore diameter distribution of Ni-YSZ fuel electrode determined by mercury porosimetry. Measurement was carried for a NiO-YSZ peace after reduction treatment at 800 °C for 2 h in 10% H_2-90% N_2. Resulting median diameter on volume basis was 0.93 µm.

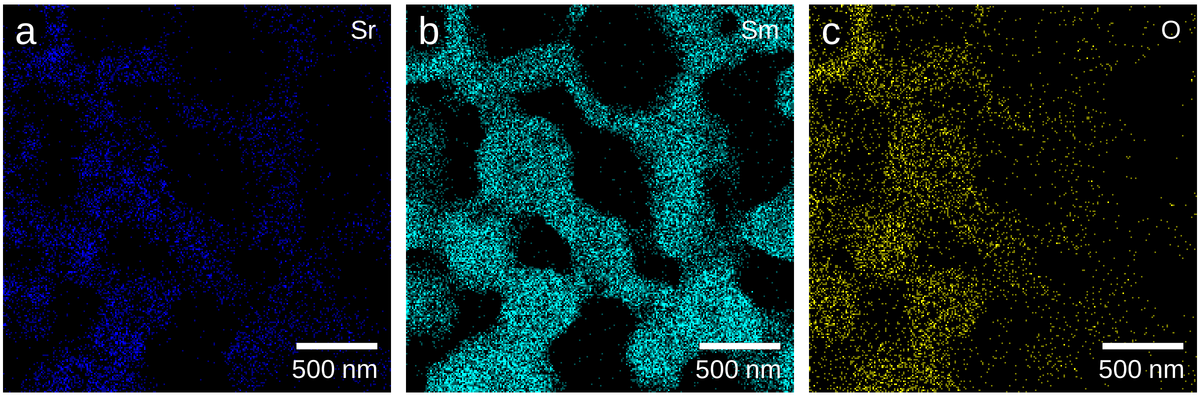

Supplementary Figure 10 Cross-sectional STEM-EDX images of SSC-SDC(50:50) nanocomposite electrode. EDX mappings of **a** Sr, **b** Sm, and **c** O.

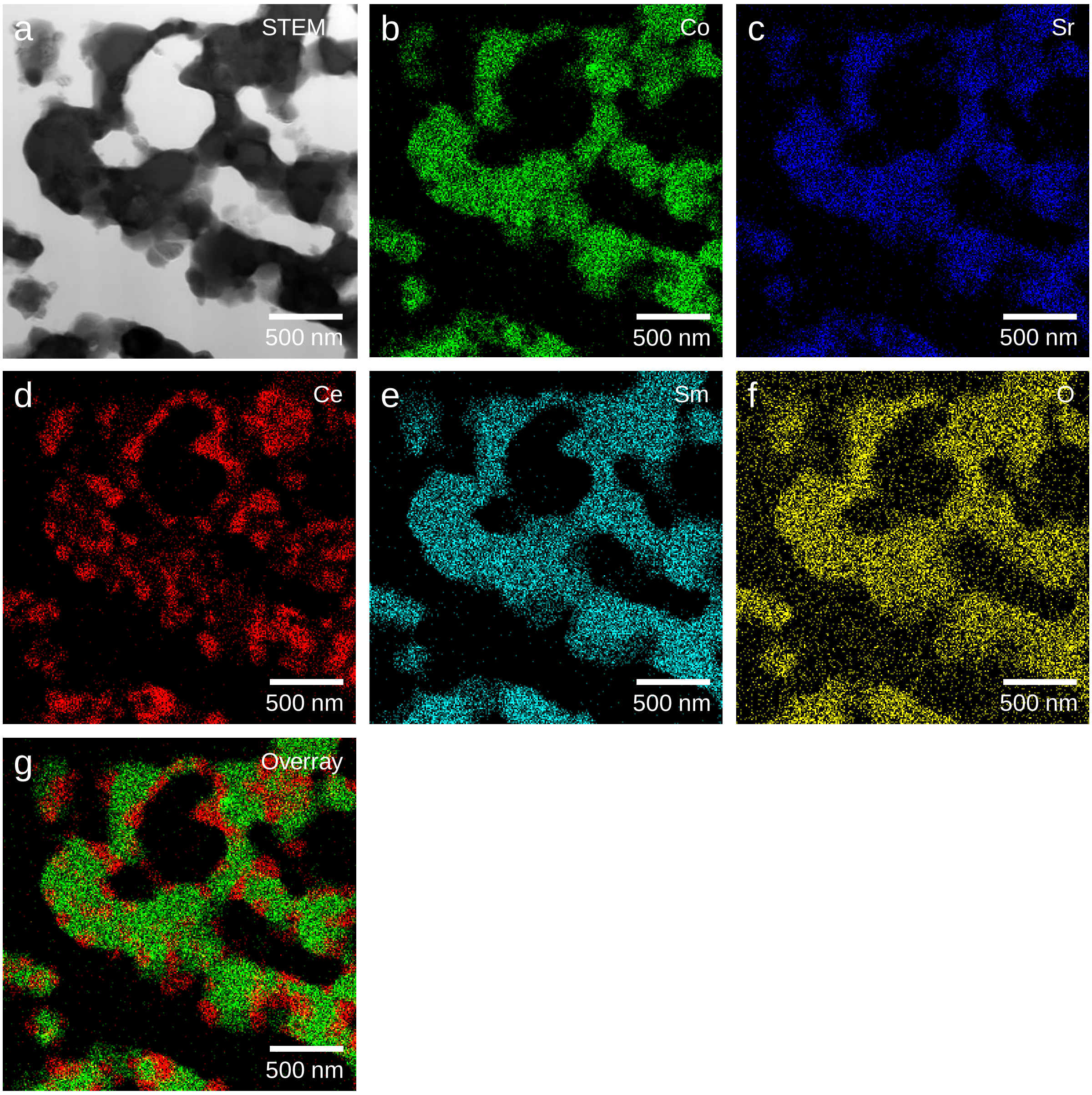

Supplementary Figure 11 Cross-sectional STEM-EDX images of SSC-SDC(70:30) nanocomposite electrode. **a** STEM image. **b** EDX mappings of Co, **c** Sr, **d** Ce, **e** Sm, and **f** O. **g** Overlay image of Co and Ce EDX mappings.

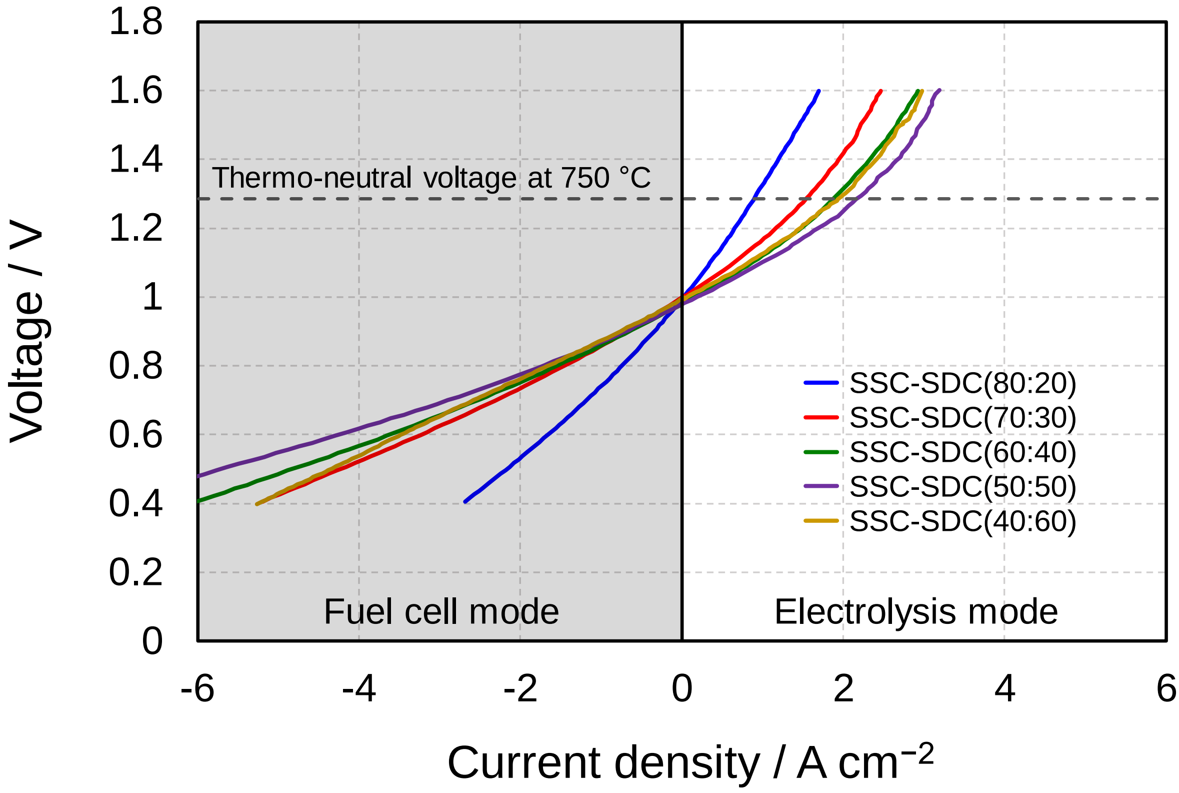

Supplementary Figure 12 *I*–*V* characteristics of SOECs with different composition ratio of SSC-SDC nanocomposite electrodes at 750 °C under 20% humidity condition. H_2O–H_2 gaseous mixture was fed to the fuel electrode and air was fed to the oxygen electrode. Both electrolysis and fuel cell modes were evaluated.

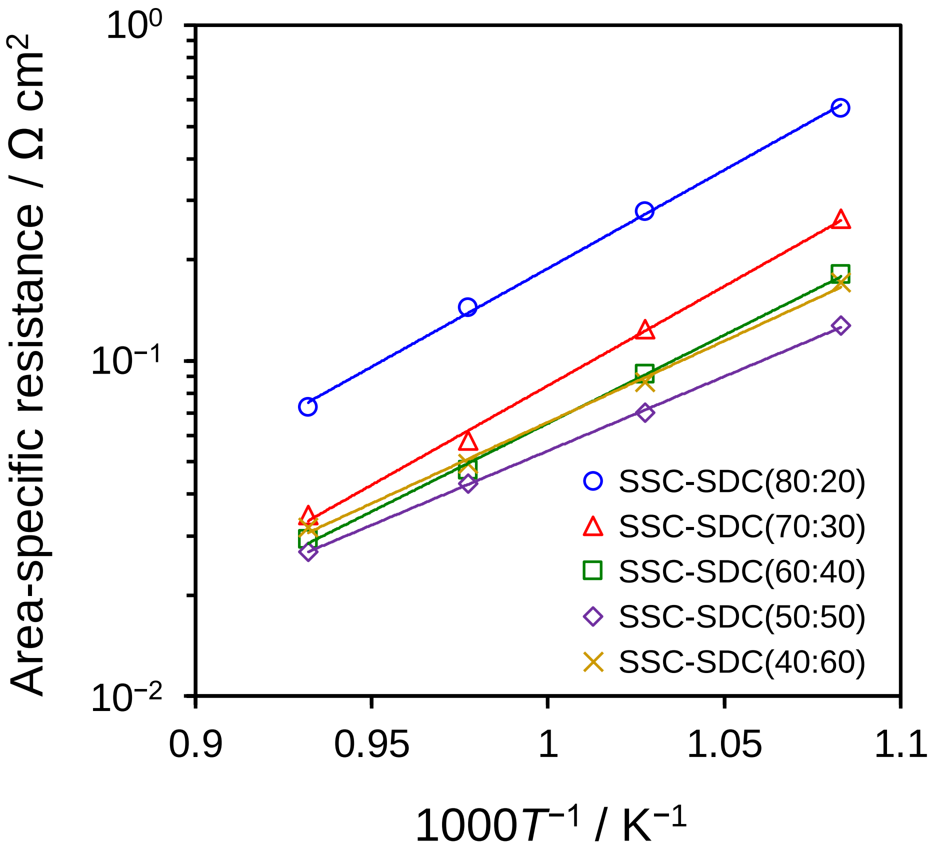

Supplementary Figure 13 Area-specific resistances (ASRs) of electrode polarization for SOECs with different composition ratio of SSC-SDC nanocomposite electrodes under 20% humidity condition. H_2O–H_2 gaseous mixture was fed to the fuel electrode and air was fed to the oxygen electrode. The ASRs were estimated based on impedance spectra under OCV condition.

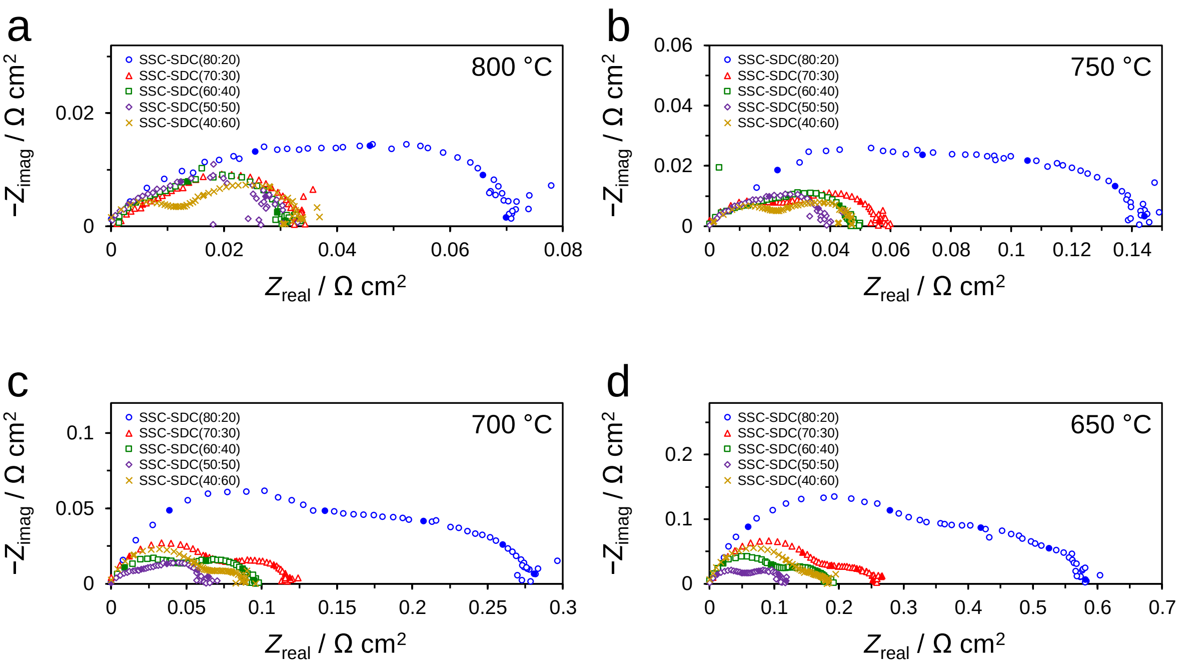

Supplementary Figure 14 Impedance spectra of SOECs with different composition ratio of SSC-SDC nanocomposite electrodes under 20% humidity condition. Measurements were carried out under OCV condition at **a** 800 °C, **b** 750 °C, **c** 700 °C, and **d** 650 °C. H_2O–H_2 gaseous mixture was fed to the fuel electrode and air was fed to the oxygen electrode.

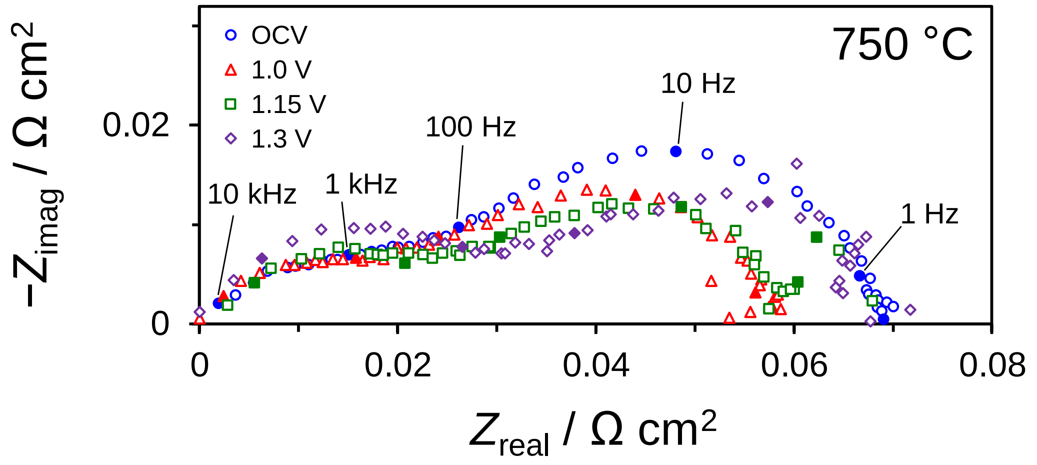

Supplementary Figure 15 Impedance spectra of SOEC with SSC-SDC(50:50) nanocomposite electrode under 50% humidity condition. Measurements were carried out at 750 °C under OCV and electrolysis operating conditions (1.0 V, 1.15 V, and 1.3 V).

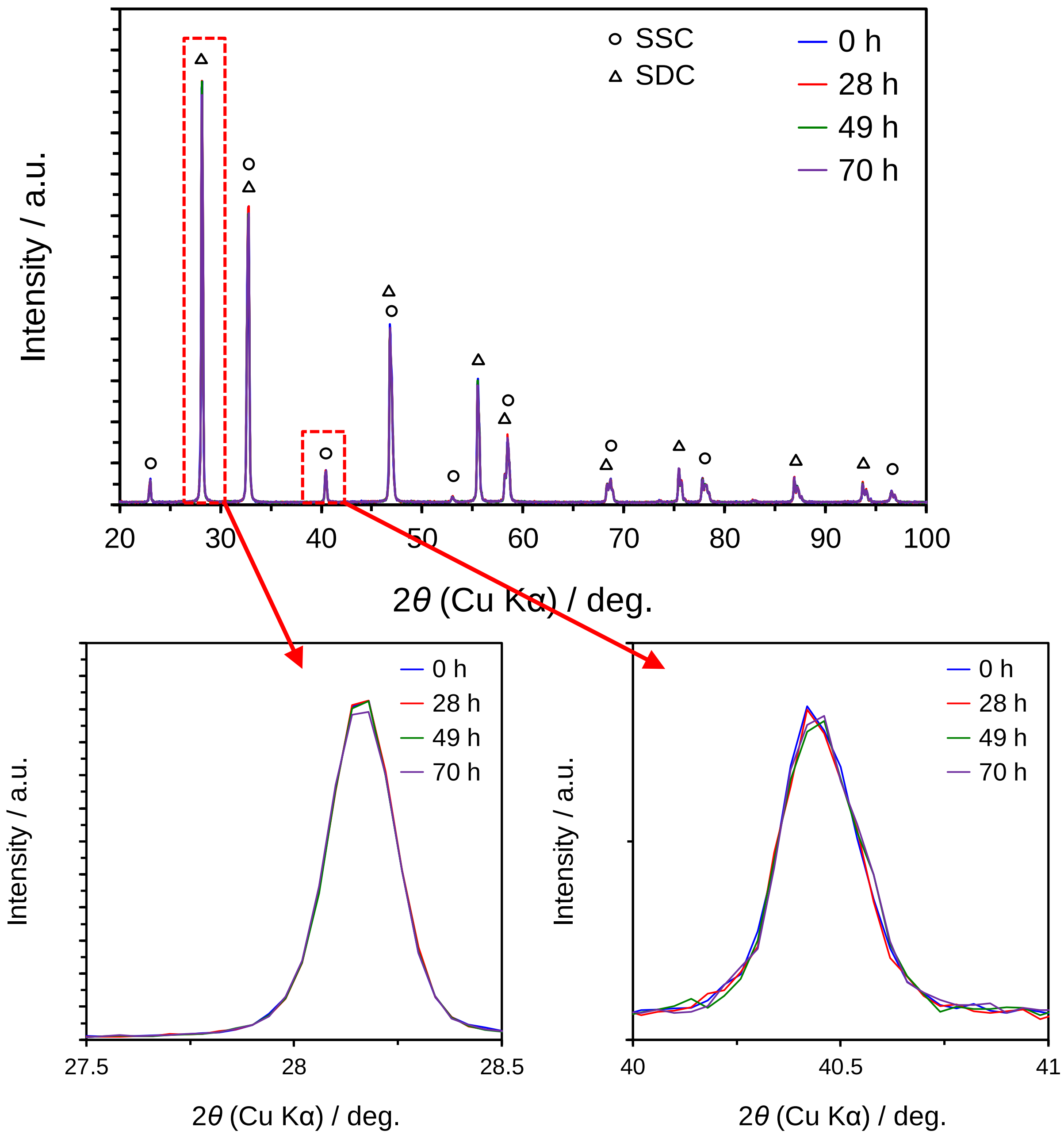

Supplementary Figure 16 High-temperature XRD results for SSC-SDC(50:50) nanocomposite electrode at 750 °C in air as a function time. The electrode (1.77-cm^2 electrode area and approximately 20-µm thick) was prepared at 950 °C for 1 h in air on a GDC disk (20-mm in diameter and 1-mm thick). The parallel-beam method was applied for the XRD measurements with Cu Kα radiation and with step size of 0.04°. The XRD patterns remained unchanged during 70 h operation.

Supplementary Table 1 Comparison in crystal size of SSC-SDC nanocomposite particles before and after sintering at 950 °C for 1 h in air.

**Sample**	**Crystal size (nm)**			
	**Before sintering**		**After sintering**	
	**SSC**	**SDC**	**SSC**	**SDC**
SSC-SDC(80:20)	13	19	40	41
SSC-SDC(70:30)	13	17	38	43
SSC-SDC(60:40)	15	15	35	43
SSC-SDC(50:50)	15	14	33	43
SSC-SDC(40:60)	15	15	33	43

Supplementary Table 2 Recent articles about solid oxide cells with EIS data under OCV and operating conditions published by Nature Publishing Group.

**Journal**	**Year**	**EIS under OCV**	**EIS under operation**	**Ref.**
Nat. Commun.	2017	Figures 2c and 5	—	1
Nat. Commun.	2019	Figure 8	—	2
Nat. Commun.	2019	Figures 2c and 2d	—	3
Nature	2016	Figure 2b	—	4
Nature	2018	Figure 1g	—	5
Nat. Mater.	2017	Figure 3	—	6
Nat. Energy	2017	Figure 5b	—	7
Nat. Energy	2018	In Method	—	8
Nat. Energy	2018	Figure 4c	—	9
Nat. Energy	2019	—	Figures 6b and 6d	10

Note that this table lists up all the studies with EIS data in "*Nat. Commun.*, *Nature*, *Nat. Mater.* and *Nat. Energy*" searched with a term of "SOFC" in 2015–2019. In the most cases, the researchers have shown EIS data under OCV condition.

Supplementary Table 3 Comparison of current densities in high-temperature steam electrolysis using SOECs with various oxygen electrodes in a temperature range of 750–850 °C at 1.3 V.

**Oxygen electrode Material**	**Measurement condition**		**Current density (A cm^{-2})**	**Ref.**
	**Temperature (°C)**	**Humidity (%)**		
SSC-SDC(50:50)	750	50	3.13	This study
SSC-SDC(50:50)	800	50	4.08	This study
(La,Sr)$MnO_{3-\delta}$	800	50	ca. 0.8	11
LSCF-GDC	850	50	ca. 0.74	12
LSCF-GDC	800	50	0.845	13
LSCF-GDC	750	50	0.9	14
SSC-infiltrated LSCF-GDC	750	50	1.8	14
SSC-infiltrated YSZ	750	50	0.71	15
$Ba_{0.5}Sr_{0.5}Co_{0.8}Fe_{0.2}O_{3-\delta}$	850	80	ca. 0.4	16
$Nd_2NiO_{4+\delta}$	750	47	0.40	17
$La_{1.7}Sr_{0.3}Co_{0.5}Ni_{0.5}O_{4.08}$	850	31	0.87	18

SSC, $Sm_{0.5}Sr_{0.5}CoO_{3-\delta}$

LSCF, $La_{0.6}Sr_{0.4}Co_{0.2}Fe_{0.8}O_{3-\delta}$

GDC, $Ce_{0.9}Gd_{0.1}O_{1.95}$

Supplementary Table 4 Operating temperatures of SSC-based electrodes in SOECs or SOFCs.

**Oxygen electrode Material**	**Electrolyte Material**	**Operating temperature (°C)**	**Ref.**
SSC-SDC (70:30 wt%)	SDC	400–600	19
SSC-SDC (75:25 wt%)	SDC	400–650	20
SSC-SDC (75:25 wt%)	SDC	500–650	21
SSC-SDC (50:50 wt%)	SDC	600	22
SSC-SDC (70:30 wt%)	SDC	550–650	23
SSC	$La_{0.9}Sr_{0.1}Ga_{0.8}Mg_{0.2}O_3$	600–800	24
$Sm_{0.6}Sr_{0.4}CoO_{3-\delta}$	$La_{0.9}Sr_{0.1}Ga_{0.8}Mg_{0.2}O_3$	400–700	25
SSC	$La_{0.9}Sr_{0.1}Ga_{0.8}Mg_{0.2}O_3$	500–700	26
SSC-infiltrated LSCF-GDC	YSZ	750	14
SSC-infiltrated YSZ	YSZ	750–850	15

SSC, $Sm_{0.5}Sr_{0.5}CoO_{3-\delta}$; SDC, $Ce_{0.8}Sm_{0.2}O_{1.9}$; LSCF, $La_{0.6}Sr_{0.4}Co_{0.2}Fe_{0.8}O_{3-\delta}$; GDC, $Ce_{0.9}Gd_{0.1}O_{1.95}$

Note that the reason why many studies have used SSC-based electrodes at lower temperatures (< 700 °C) is related to the electrolyte material. As shown in Supplementary Table 4, SSC-based electrodes are often used with ceria-based electrolytes due to good chemical compatibility. Because ceria-based electrolytes show electron conductivity (current leakage) above 650 °C, these types of solid oxide cells with SSC-based electrodes have been operated below 650 °C[19–23]. In the cases using other electrolytes, such as zirconia-based electrolytes with GDC interlayer and $LaGaO_3$-based electrolytes, SSC electrodes have been operated in a higher temperature range of 700–850 °C[14,15,24–26].

Supplementary Table 5 The amount of used nitrate reagents to prepare nitrate solutions (1000 mL) for spray pyrolysis.

**Sample**	**Nitrate reagent (g)**			
	**$Co(NO_3)_2·6H_2O$**	**$Sr(NO_3)_2$**	**$Ce(NO_3)_3·6H_2O$**	**$Sm(NO_3)_3·6H_2O$**
SSC-SDC(80:20)	22.022	8.007	8.452	18.979
SSC-SDC(70:30)	18.761	6.821	12.344	17.485
SSC-SDC(60:40)	15.668	5.697	16.036	16.068
SSC-SDC(50:50)	12.730	4.629	19.543	14.722
SSC-SDC(40:60)	9.935	3.612	22.879	13.441

Supplementary References

1. Li, M., Zhao, M., Li, F., Zhou, W., Peterson, V. K., Xu, X., Shao, Z., Gentle, I. and Zhu, Z. A niobium and tantalum co-doped perovskite cathode for solid oxide fuel cells operating below 500 °C. *Nat. commun.* **8**, 13990 (2017).
2. Li, T., Heenan, T. M. M., Rabuni, M. F., Wang, B., Farandos, N. M., Kelsall, G. H., Matras, D., Tan, C., Lu, X., Jacques, S. D. M., Brett, D. J. L., Shearing, P. R., Michiel, M. D., Beale, A. M., Vamvakeros, A. and Li, K. Design of next-generation ceramic fuel cells and real-time characterization with synchrotron X-ray diffraction computed tomography. *Nat. commun.* **10**, 1497 (2019).
3. Xia, C., Mi, Y., Wang, B., Lin, B., Chen, G. & Zhu, B. Shaping triple-conducting semiconductor $BaCo_{0.4}Fe_{0.4}Zr_{0.1}Y_{0.1}O_{3-\delta}$ into an electrolyte for low-temperature solid oxide fuel cells. *Nat. commun.* **10**, 1707 (2019).
4. Zhou, Y., Guan, X., Zhou, H., Ramadoss, K., Adam, S., Liu, H., Lee, S., Shi, J., Tsuchiya, M., Fong, D. D. and Ramanathan, S. Strongly correlated perovskite fuel cells. *Nature.* **534**, 231–234 (2016).
5. Duan, C., Kee, R. J., Zhu, H., Karakaya, C., Chen, Y., Ricote, S., Jarry, A., Crumlin, E. J., Hook, D., Braun, R., Sullivan, N. P. and O'Hayre, R. Highly durable, coking and sulfur tolerant, fuel-flexible protonic ceramic fuel cells. *Nature.* **557**, 217–222 (2018).
6. Rupp, G. M., Rupp, G. M., Opitz, A. K., Nenning, A., Limbeck, A. and Fleig, J. Real-time impedance monitoring of oxygen reduction during surface modification of thin film cathodes. *Nat. Mater.* **16**, 640–645 (2017).
7. Shin, J. F., Xu, W., Zanella, M., Dawson, K., Savvin, S. N., Claridge, J. B. and Rosseinsky, M. J. Self-assembled dynamic perovskite composite cathodes for intermediate temperature solid oxide fuel cells. *Nat. Energy* **2**, 16214 (2017).
8. Chen, Y., Glee, B. Tang, Y., Wang, Z., Zhao, B., Wei, Y., Zhang, L., Yoo, S., Pei, K., Kim,

J. H., Ding, Y., Hu, P. Tao, F. F. and Liu, M. A robust fuel cell operated on nearly dry methane at 500 °C enabled by synergistic thermal catalysis and electrocatalysis. *Nat. Energy* **3**, 1042–1050, (2018).

9. Choi, S., Kucharczyk, C. J., Liang, Y., Zhang, X., Takeuchi, I., Ji, H.-I. and Haile, S. M. Exceptional power density and stability at intermediate temperatures in protonic ceramic fuel cells. *Nat. Energy* **3**, 202–210, (2018).
10. Duan, C., Kee, R., Zhu, H., Sullivan, N., Zhu, L., Bian, L., Jennings, D. and O'Hayre, R. Highly efficient reversible protonic ceramic electrochemical cells for power generation and fuel production. *Nat. Energy* **4**, 230–240, (2019).
11. Brisse, A., Schefold, J. and Zahid, M. High temperature water electrolysis in solid oxide cells. *Int. J. Hydrogen Energy* **33**, 5375–5382 (2008).
12. Laurencin, J., Hubert, M., Sanchez, D. F., Pylypko, S., Morales, M., Morata, A., Morel, B., Montinaro, D., Lefebvre-Joud, F. and Siebert, E. Degradation mechanism of $La_{0.6}Sr_{0.4}Co_{0.2}Fe_{0.8}O_{3-\delta}/Gd_{0.1}Ce_{0.9}O_{2-\delta}$ composite electrode operated under solid oxide electrolysis and fuel cell conditions. *Electrochim. Acta* **241**, 459–476 (2017).
13. López-Robledo, M. J., Laguna-Bercero, M.A., Larrea, A. and Orera, V. M. Reversible operation of microtubular solid oxide cells using $La_{0.6}Sr_{0.4}Co_{0.2}Fe_{0.8}O_{3-\delta}$-$Ce_{0.9}Gd_{0.1}O_{2-\delta}$ oxygen electrodes. *J. Power Sources* **378**, 184–189 (2018).
14. Lee, S., Kim, J., Son, J.-W., Lee, J.-H., Kim, B.-K., Je, H.-J., Lee, H.-W., Song, H. and Yoon, K. J. High performance air electrode for solid oxide regenerative fuel cells fabricated by infiltration of nano-catalysts. *J. Power Sources* **250**, 15–20 (2014).
15. Zhang, Y. Han, M. and Sun, Z. High performance and stability of nanocomposite oxygen electrode for solid oxide cells. *Int. J. Hydrogen Energy* in press.
16. Zhang, W., Yu, B. and Xu, J. Investigation of single SOEC with BSCF anode and SDC barrier layer. *Int. J. Hydrogen Energy* **37**, 837–842 (2012).

17. Chauveau, F., Mougin, J., Bassat, J. M., Mauvy, F. and Grenier, J. C. A new anode material for solid oxide electrolyser: The neodymium nickelate $Nd_2NiO_{4+\delta}$. *J. Power Sources* **195**, 744–749 (2010).

18. Laguna-Bercero, M. A. Recent advances in high temperature electrolysis using solid oxide fuel cells: A review. *J. Power Sources* **203**, 4–16 (2012).

19. Xia, C., Rauch, W. Chen, F. and Liu, M. $Sm_{0.5}Sr_{0.5}CoO_3$ cathodes for low-temperature SOFCs. *Solid State Ionics* **149**, 11–19 (2002).

20. Zhang, X., Robertson, M., Yick, S., Deĉes-Petit. C., Styles, E., Qu, W., Xie, Y., Hui, R., Roller, J., Kesler, O., Maric, R. and Ghosh, D. $Sm_{0.5}Sr_{0.5}CoO_3$ + $Sm_{0.2}Ce_{0.8}O_{1.9}$ composite cathode for cermet supported thin $Sm_{0.2}Ce_{0.8}O_{1.9}$ electrolyte SOFC operating below 600 °C. *J. Power Sources* **160**, 1211–1216 (2006).

21. Zhang, X., Zhang X., Robertson, M., Deĉes-Petit. C., Qu, W., Kesler, O., Maric, R. and Ghosh, D. Internal shorting and fuel loss of a low temperature solid oxide fuel cell with SDC electrolyte. *J. Power Sources* **164**, 668–677 (2007).

22. Chen, H., Cheng, K., Ye, F. and Weng, W. Preparation and characterization of graded SSC–SDC MIEC cathode for low-temperature solid oxide fuel cells. *Ceram. Int.* **37**, 1209–1214 (2011).

23. Sun, H., Zhang, Y., Gong, H., Li, Q., Bu, Y. and Li, T. Anode-supported SOFCs based on $Sm_{0.2}Ce_{0.8}O_{2-\delta}$ electrolyte thin-films fabricated by co-pressing using microwave combustion synthesized powders. *Ceram. Int.* **42**, 4285–4289 (2016).

24. Ishihara, T., Honda, M., Shibayama, T., Minami, H., Nishiguchi, H. and Takita, Y. Intermediate temperature solid oxide fuel cells using a new $LaGaO_3$ based oxide ion conductor. *J. Electrochem. Soc.* **145**, 3177–3183 (1998).

25. Yan, J., Matsumoto, H., Enoki, M. and Ishihara, T. High-power SOFC using $La_{0.9}Sr_{0.1}Ga_{0.8}Mg_{0.2}O_{3-\delta}/Ce_{0.8}Sm_{0.2}O_{2-\delta}$ composite film. *Electrochem. Solid-State Lett.* **8**,

A389–A391 (2005).

26. Ju, Y.-W., Eto, H., Inagaki, T., Ida, S. and Ishihara, T. Preparation of Ni–Fe bimetallic porous anode support for solid oxide fuel cells using $LaGaO_3$ based electrolyte film with high power density. *J. Power Sources* **195**, 6294–6300 (2010).
